# Reviews

**Published:** 1880-07

**Authors:** 


### REVIEWS.

---

**Post-mortem Examinations with Especial Reference to Medico-legal Practice.** By RUDOLPH VIRCHOW, of Berlin Charité Hospital. Translated from the second German edition, by Dr O P. Smith. Philade'phia : Presley Blakiston, 1012 Walnut St , 1880.

A little book of one hundred and forty pages, 16 mo. with a number of illustrations. Prof. Virchow's early experiences as prosector in the morgue of the Berlin Charity Hospital are given, as well as the subsequent history showing the present system of examining the bodies of the dead. He also gives the regulations at present in force throughout Germany, "for the guidance of medical jurists in performing autopsies and drawing up reports." As the translator remarks: "It is much to be wished that a method similar to the one which has received the high sanction of Prof. Virchow were adopted in this country." Although no better than Delafield on post-mortems, it is a book well worth possessing especially by those who have not already one or more recent works upon the subject. V. .P

---

**The Pharmacopœia of the British Hospital for Diseases of the Skin.** London, 2d Edition Edited by BALMANNO SQUIRE, M.B., Senior Surgeon to the Hospital. London: J. & A. Churchill.

By the kindness of the author we have received this little work. It contains, as the title indicates, the formulae of the special preparations used in this great hospital. Although we find, in looking over its 76 pages, almost nothing not in common use in this city, the book is of value as a compilation of well-tried and approved formulæ. D.

---

**A Practical Hand Book of Medical Chemistry applied to Clinical Research and the Detection of Poison.** By WILLIAM H. GREENE, M. D, Demonstrator of Chemistry, University of Pennsylvania. Philadelphia: H. C. Lea's Son & Co.

The absolute necessity to the medical man of some knowledge of medical chemistry is conceded, but in the nature of things it is impossible that a practicing physician should be a chemist. Having, however, that general knowledge of the science without which in this day he ought not to hold a diploma, it is easy with the aid of a good hand book to acquire an acqaintance with these compounds which normally form part of our tissues, and to detect substances which are themselves the evidence of pathological action.

This is the object of Dr. Greene's book, and he has succeeded in giving to the profession a useful work. The first part of the book is devoted to a brief consideration of the organic proximate principles taking part in the animal economy; the second part to the analysis of secretions, excretions, etc.; while the third part treats of the detection of poisons, only so far as is of value and interest to physicians. The book is well arranged, and likely to be a popular and useful one to medical men. D.

---

**A Text Book of Physiology.** By M. Foster, M. A, M. D, F. R. S. Prælector in Physiology and Fellow of Trinity College, Cambridge. From the 3d and revised English edition, with notes and additions. By Ed. T. Reichert, Demonstrator of Experimental Therapeutics, University of Pennsylvania, with two hundred and fifty-nine Illustrations. Philadelphia: H. C. Lea's Son & Co, 1880.

Foster's Physiology, English Edition, has been, in our humble opinion, the best work on the subject in the language for the advanced student. The author however presupposed an acquaintance with the details of physiological anatomy on the part of his readers, and this while a convenience to a certain class, has proved a serious drawback to the use of the work as a text book for use in our schools. We have therefore looked forward to the appearance of an American edition, with the hope that under the able editorship of Dr. Reichert, this deficiency might be supplied. The book before us surpasses our expectations. Nothing has been omitted from the last English edition, but about one hundred and forty pages of additions have been made by the editor, in most cases with much conciseness and much good judgment. The illustrations have been increased from seventy-two to two hundred and fifty-nine. The work itself is above criticism. The ripened intellect of the author weighs with laborious minuteness the investigations of physiologists past or present, sifts out truth from error, and discusses conflicting theories with a fairness, fullness and conciseness which lends freshness and vigor to the entire book. Such enormous advances have been made recently in our physiological knowledge, that it is safe to say that physicians whose libraries contain works on this subject issued ten, nay, even five years ago, are behind the times. Every man who wishes to march in the vanguard of his profession should possess this book, and by all means buy the American edition. The book contains nearly 1,100 pages. The publishers' work is well done. D.

---

**The Venereal Diseases, including Stricture of the Male Urethra.** By E. L. KEYES, A. M., M. D. New York: William Wood & Co. 1880.

This work is an octavo volume of three hundred and fifty pages, one of Wood's Medical Library Series. The writer is well known as the author of a number of papers upon the so-called tonic treatment of syphilis. In connection with Dr. Van Buren he has also written a standard work upon Venereal Diseases. The present work is especially noted for being concise and practical. In the author's words, it has been his aim "to present the various venereal diseases as clearly as possible, avoiding such unnecessary refinement upon theoretical and mooted points as would be apt to lead to confusion or to error. Practical utility, as well as what I believe to be sound doctrine, has been kept constantly in view. . . ." That the author is thoroughly qualified to write upon venereal diseases, is too well known to need affirming; but if such were needed, this work would be a sufficient proof. Especially to those general practitioners who desire a standard work essentially practical, do we recommend this volume. V. P.

---

**The Therapeutics of Gynecology and Obstetrics.** By WILLIAM B. ATKINSON, A. M., M. D. Philadelphia: D. G. Brinton, 115 South Seventh St., 1880.

This book of three hundred and fifty pages is one similar in purpose and character to the preceding, but, as is evident from the title, is more limited in scope. To our mind it contains a large number of good prescriptions, these have been obtained from the most eminent gynecologists of this and other countries. The work cannot fail to be a great aid to those who do not happen to be specialists. V. P.

